# Numerical Study on the Shear Behavior of a Late-Model Cold-Formed Stainless Steel C-Shaped Beam

**DOI:** 10.3390/ma18010091

**Published:** 2024-12-28

**Authors:** Shuang-E Huangfu, Zhong Tao, Zhenglin Zhang, Zihao Wang, Ji Zhang

**Affiliations:** 1 Faculty of Civil Engineering and Mechanics, Kunming University of Science and Technology, Kunming 650500, China; 2 City College, Kunming University of Science and Technology, Kunming 650051, China; 3 Yunnan Seismic Engineering Technology Research Center, Kunming University of Science and Technology, Kunming 650500, China; 4 CCFED The Fifth Construction & Engineering Co., Ltd., Shenzhen 518052, China; 5 The Third Construction Co., Ltd. of China Railway Construction Engineering Group, Tianjin 300451, China; 6 CCCC First Highway Consultants Co., Ltd., Xi’an 710075, China

**Keywords:** folded flange, stainless steel, stiffening web, parametric research, shear capacity

## Abstract

The failure mode of thin-walled C-channel beams typically manifests as premature local buckling of the compression flange, leading to insufficient utilization of material strength in both the flange and the web. To address this issue, this study adopts the approach of increasing the number of bends to reinforce the flange and adding V-shaped stiffeners in the middle of the web to reduce the width-to-thickness ratio of the plate elements, thereby delaying local buckling and allowing for greater plastic deformation. However, the challenge lies in the irregular cross-sectional shape and complex buckling patterns. Therefore, this paper aims to explore a suitable cross-sectional form to expand the application of stainless steel members. Subsequently, three-point bending tests were conducted on the optimally designed stainless C-channel beam with folded flanges and mid-web stiffeners. The finite element simulation results were compared and analyzed with the experimental results to validate the model’s effectiveness. After verifying the correctness of the finite element model, this study conducted numerical parameterization research to investigate the effects of the shear span ratio, complex edge stiffeners, web height–thickness ratio, and V-shaped stiffener size on the shear performance of stainless steel folded flange C-beams. The results show that changing the shear span ratio has a significant impact on the shear capacity and vertical deflection deformation of components; increasing the web height–thickness ratio can enhance the shear capacity of the component; elevating the V-shaped stiffener size can slightly improve the shear capacity of components; and for the stainless steel C-shaped beam with folded flanges and intermediate stiffening webs, adding edge stiffeners cannot remarkably promote the shear capacity of the component.

## 1. Introduction

Cold-formed thin-wall steels have attracted widespread attention in engineering circles because of their lightweight, high-strength, diversity in profile manufacturing, and versatility [1,2]. With the research of global scholars, cold-formed thin-wall components with various cross-sections have been applied to building structures. However, studies on cold-formed stainless steel components are rare. C-shaped beams are a typical cold-formed thin-wall stainless steel component commonly used for roof purlins and wall beams [3,4]. Assuming overall stability is ensured, the failure mode of purlins typically manifests as local buckling of the compression flange. Due to premature local buckling of the flange, the material strength of both the flange and the web is not fully utilized [5,6]. Due to the characteristics of small thickness, large width–thickness ratio, and open sections, this cross-sectional component has complicated buckling performance [7,8,9]. Therefore, studying efficient cross-sectional profiles and beam–column connections is of great significance for improving seismic performance [10,11], preventing buckling [12], and expanding the application range of stainless steel members.

Recently, numerous approaches have emerged to reinforce thin-walled steel members [13,14], innovate materials [15], and enhance load-bearing capacity and ductility. It is worth noting that stainless steel material features strong corrosion resistance, aesthetic appeal, long service life, and low maintenance costs, inherently making it a high-performance, environmentally friendly building material [16]. Given the relatively high initial investment cost of stainless steel, significant improvements in member performance have been achieved through the optimization of cross-sectional shapes and properties [17]. Compared to the original channel steel section, C-channel members with bent flanges exhibit higher strength, stiffness, and ductility [18]. However, this cross-sectional design is limited to point-to-surface contact, complicating its connection to floor slabs [19]. Therefore, developing a more practical folded-flange section not only facilitates connection to floor slabs but also allows for flexible adjustment according to the internal space of the module. During earthquakes, the flange portion can undergo plastic deformation to a certain extent, dissipating seismic energy [20,21], thus meeting the demands of modern construction. The optimized folded flange section, compared to the traditional double C-section without edge stiffeners, increases the ultimate flexural bearing capacity by 57% [22].

In recent years, extensive research has been conducted on the flexural performance of the original C-channel beam [23,24]. Pham et al. [23] investigated the shear performance of cold-formed C-channel beams with and without edge stiffeners, revealing that flanges significantly influence the shear capacity of C-channel beams. Particularly for sections with narrow flanges, members are prone to premature buckling in torsional and lateral buckling modes due to insufficient lateral support. Although some scholars have examined the structural performance of austenitic stainless steel channel sections under axial loads [25], concluding that this material exhibits excellent load-bearing properties, research on shear behavior remains limited. Previous studies have been confined to examining web shear buckling without considering the performance of the entire cross-section (including flanges) [26], lacking research on full-section buckling of thin-walled profiles under shear. Therefore, it is crucial to explore the effect of adjacent plate elements on shear buckling stress and understand the buckling patterns of thin-walled steel sections (rather than flat plate elements).

However, research on stiffened C-channel beams remains relatively limited. Notably, the most popular longitudinal intermediate stiffeners [27,28,29,30] have been employed to strengthen webs to improve their deformation resistance [31]. Dissanayake et al. [32] performed a numerical study on the shear performance of cold-formed stainless steel stiffening web channel steels. They believed that the depth of the web stiffener had a profound impact on the shear buckling capacity of the channel steel section.

This paper aims to modify the traditional channel section of steel by adopting a C-channel section with folded flanges (i.e., increasing the number of bends to reinforce the flange) composed of inclined and flat flanges, and adding V-shaped stiffeners in the middle of the web to reduce the width-to-thickness ratio of plate elements, thereby delaying local buckling and allowing for greater plastic deformation. In subsequent tests, we conduct flexural performance tests on members with various cross-sectional shapes and stiffener positions to enhance the practicality and effectiveness of this optimized cross-section in real-world applications. The objective of this study is to explore a suitable cross-sectional form, aiming to provide a theoretical foundation for future research on various buckling modes of steel structural members with complex cross-sectional shapes. Firstly, cross-sectional dimensions were designed in accordance with specifications to ensure compliance with the limitations of width–thickness ratio. Subsequently, three-point bending tests were performed on stainless C-channel beams with folded flanges and mid-web stiffeners. Finally, the finite element simulation analysis method was used to explore the deformation and bearing capacity and the effects of various conditions on the shear capacity and failure mode of the C-shaped beam under the conditions of changing the shear span ratio, adding complex edge stiffeners, the web height–thickness ratio, and the V-shaped stiffener size of the C-shaped beam.

## 2. Experimental Overview

### 2.1. Sample Design

**Figure 1:**
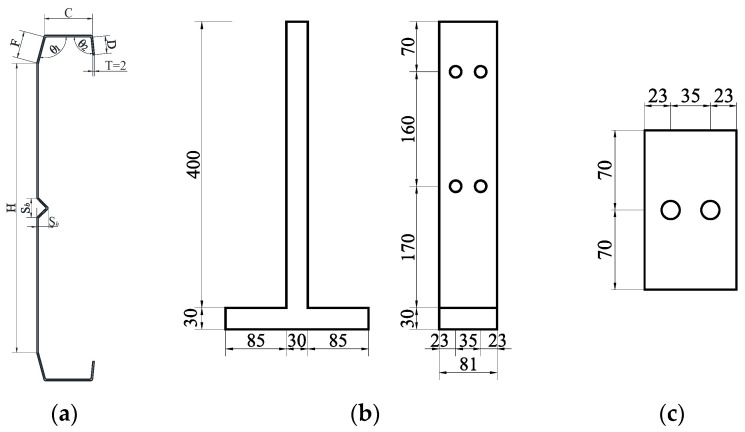
Schematic diagram of the parameters of components and connectors. (**a**) C-shaped beam, (**b**) T-shaped connecting board, and (**c**) web stiffener plate.

This study selects the optimal section shape according to the EC3 [33] specification for the plate angle, width–thickness ratio, and relative size of each component of the section. As shown in Figure 1a, the height of the web is H = 320 mm, the width of the bevel flange is F = 30 mm, the width of the flat flange is C = 50 mm, and the curling width is D = 20 mm. The opening width and height of the V-shaped stiffener in the middle of the web are S*_b_* = 20 mm and S*_h_* = 10 mm, respectively. The inner angle of the folded flange is θ_1_ = 105°, the outer angle is θ_2_ = 95°, the V-shaped angle is θ = 90°, the cross-sectional thickness is T = 2 mm, and the bending inner diameter at the intersection of the plate is r = 2 mm. Figure 1c shows the parameters of the web stiffener plates, which are connected with M16 high-strength bolts.

**Figure 2:**
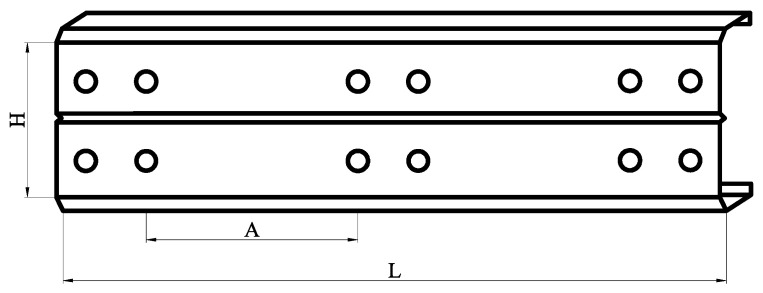
Schematic diagram of the shear span ratio parameters.

As shown in Figure 2, the shear span is A = 640 mm (A represents the spacing between the centers of the bolts on the inner side of the web). The existing literature has suggested that when the shear span ratio of a beam is less than 2, the beam is mainly controlled by the shear capacity. Therefore, this study selects a C-shaped beam with a shear span ratio K = A/H of 2 for testing. The specimens in this study were uniformly designated with four parameter variables: shear span ratio K, shear span A, web height H, and thickness T, that is, K2A640 × H320 × T2 (the parameters of the bevel and flat flanges have fixed values, and the other components are named in the same way). The measured dimensions from the experiments are listed in Table 1.

### 2.2. Material Properties

**Figure 3:**
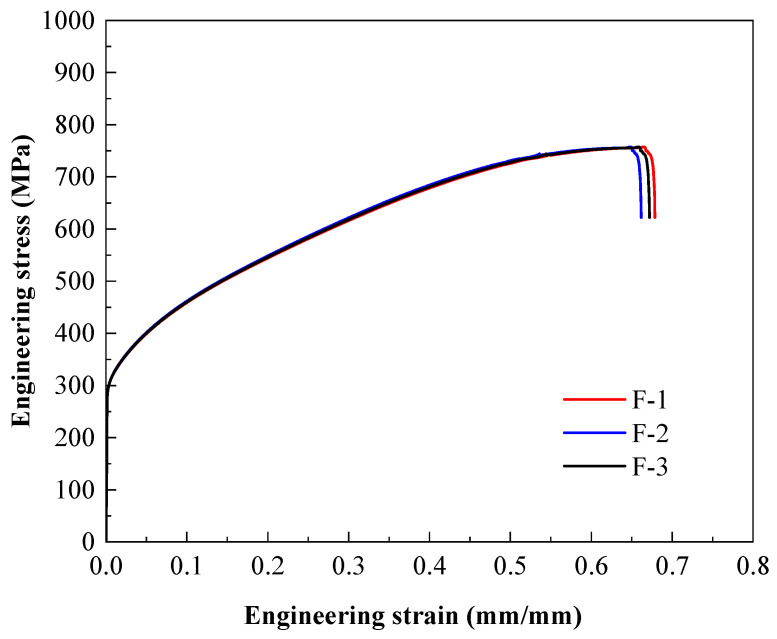
Stress–strain curves of specimens.

The material tensile specimens were cut from stainless steel components by a laser cutting machine. Three specimens were taken from the component web, tensile flange, and compressed flange, respectively. An electronic universal testing machine was employed for the tensile test. The test data were collected using an extensometer and strain collection instrument. The average values of elastic modulus, nominal yield strength, ultimate tensile strength, and elongation after fracture are 1.94 × 10^5^ MPa, 315 MPa, 757 MPa, and 48%, respectively. The stress–strain curves of the three groups of specimens are detailed in Figure 3.

### 2.3. Loading Scheme

**Figure 4:**
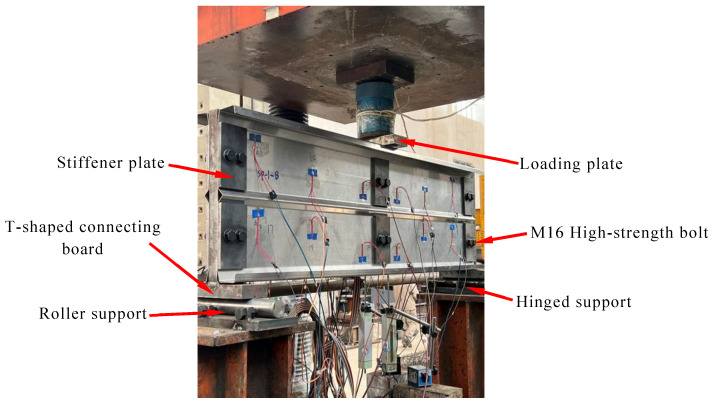
Experiment setup.

This study consulted the experimental method of Keerthan et al. [34] on the shear performance of cold-formed channel steel beams. To prevent torsion in cold-formed and thin-walled C-channel beams during bending tests, it is essential to ensure that the applied vertical load passes through the shear center of the cross-section. Typically, the shear center of these members is located outside the web plane. Prior to specimen fabrication, CUFSM software (V5.01) [35] was used to analyze the characteristics of the C-channel section, determining the position of the shear center for each cross-section. During the test, two C-channel specimens with folded flanges, exhibiting identical cross-sections, were placed back-to-back, leaving a gap equal to twice the distance from the web to the shear center between them. In Figure 4, by placing T-shaped plates and steel plates between the two specimens, the vertical load was directed through the shear center. Considering the requirements for the high-strength bolt nominal diameter, bolt hole spacing, edge distance, and end distance, as well as the web height specifications in the Standard for Design of Steel Structures [36], M16 high-strength bolts were used to connect the specimens with the T-shaped plates and steel plates, thus avoiding the impact of welding on the specimens. As the web height of the specimens gradually increased, the number of bolts correspondingly increased. Subsequent designs incorporated multiple rows of bolt holes to facilitate multiple uses of the T-shaped plates. The dimensions of contact surface dimensions for the T-shaped plate with the loading point and support points were set to 200 mm × 81 mm × 30 mm (as shown in Figure 1b). This prevents excessive displacement and slippage during testing that could arise from an overly narrow contact surface. Considering that the T-shaped plates need to be reused in multiple tests, the 30 mm thickness is sufficient to prevent deformation from repeated compression. Additionally, to prevent local buckling of the specimen’s web due to concentrated forces at the loading point and supports, 10 mm thick steel plates were added to the outer side of the connection between the specimen and the steel plate for reinforcement, as shown in Figure 1c.

Before formal loading, pre-load was used to eliminate gaps generated during the assembly process, and the value of the test jack was adjusted to zero. The test load was applied at a rate of 1 mm/min starting from scratch. Meanwhile, the test was observed in the control room. When the load dropped to 80% of the ultimate bearing capacity, the machine was manually controlled to stop loading.

## 3. Experiment Results and Analysis

### 3.1. Failure Characteristics

**Figure 5:**
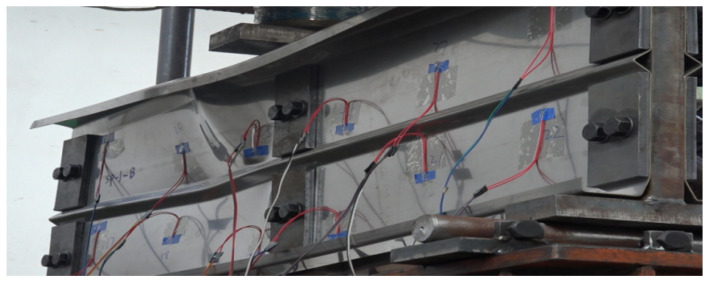
Failure characteristics of the specimens.

The failure characteristics of the specimens in this test are shown in Figure 5. The entire test process is as follows: ① In the initial stage of loading, local buckling occurs at the midspan compressed flange, and the web plate has no obvious deformation. ② When the load is about 70% of the ultimate bearing capacity, the right web of the component exhibits significant buckling deformation, showing an upward trend. ③ Continuing to load until the ultimate bearing capacity stage, the buckling deformation gradually increases and develops upwards from the midspan in the direction of 45°. ④ The test stops when the load drops to 80%, the deformation remains enlarging, and the bearing capacity declines sharply.

Based on Figure 5, the specimen failure primarily manifests as a shear failure characterized by diagonal-tension failure of the beam web, i.e., shear buckling failure [37]. The bolts between the beam web and the stiffener did not exhibit significant deformation, while warping deformation occurred in the upper part of the buckled area, including the flange and edge stiffeners. This indicates that while the web primarily bears the shear force, the flange also contributes to carrying a portion of the shear load.

### 3.2. Shear Force–Midspan Vertical Displacement Curve

**Figure 6:**
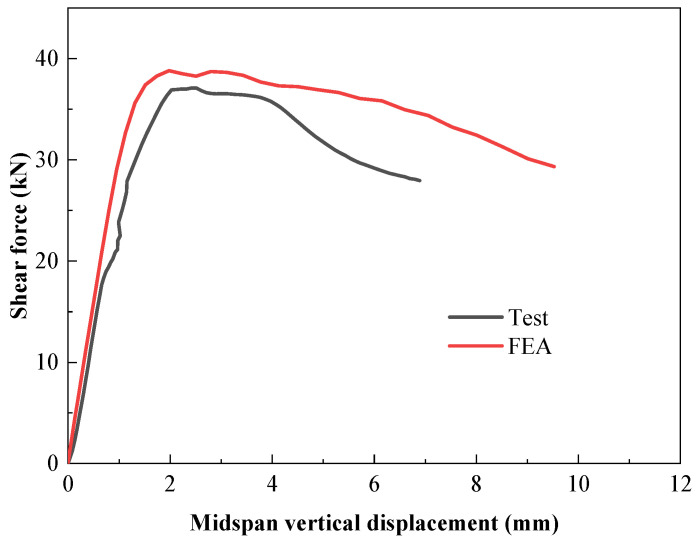
Comparison of the experiment and finite element shear force–midspan vertical displacement curves.

The shear force–midspan vertical displacement curve in Figure 6 illustrates the following: ① In the early stage of loading, the midspan vertical displacement of the specimen rises with the load. ② The applied displacement load gradually decreases after reaching the peak value, followed by a further increase in the midspan vertical displacement until the specimen fails. ③ When the load approaches the extreme point, its growth rate is relatively slow, while the midspan vertical displacement of the specimen grows rapidly, presenting outstanding ductility.

Analysis of the load–displacement curve reveals that the C-channel beam can continue to bear load after buckling occurs, and due to the influence of tensile stress, the strength of the C-channel beam significantly increases post-buckling [38]. Following the buckling of the C-channel beam, there is no abrupt decrease in the beam’s load and displacement, demonstrating excellent ductility.

## 4. Finite Element Analysis

### 4.1. Model Establishment

**Figure 7:**
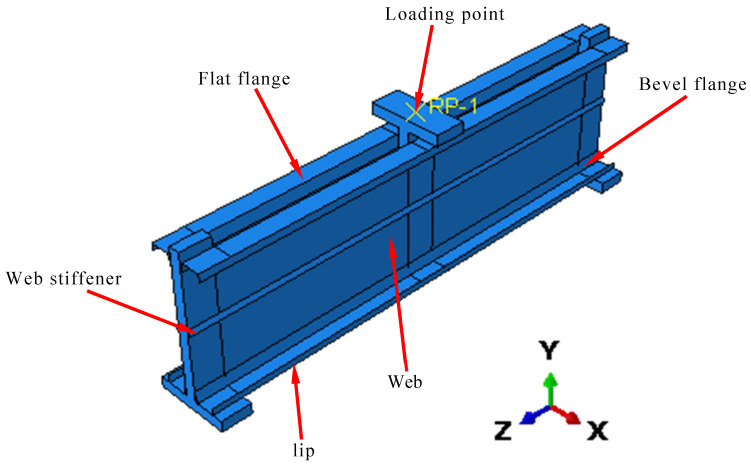
Finite element model.

This study adopted the Abaqus software (V2022) for the model. The C-shaped beam with folded flanges was stimulated using shell elements, and the T-shaped connecting board was stimulated with solid elements. Abaqus part commands were adopted for 3D sketch stretch modeling. After assembly, a geometric model consistent with the experiment was formed, as shown in Figure 7.

The C-shaped beam in the finite element model was meshed using S4R shell elements. C3D8R elements were employed for the grid division of the T-shaped connecting board. S4R shell elements have been proven to effectively simulate the nonlinear behavior of thin-wall components [39,40,41,42]. After reviewing the literature [43,44] and conducting simulation calculations, the mesh sizes of the V-shaped stiffener, corners, and curling edges of the C-shaped beam were 15 mm. For the mesh sensitivity analysis, we assumed that the geometry, material properties, and boundary conditions were accurately defined and independent of mesh refinement. Gradually reducing mesh sizes below 15 mm improved accuracy but significantly increased computational time. In contrast, mesh sizes exceeding 15 mm led to inaccurate simulation of stress concentration areas. We found that using a 20 mm mesh size for other plate elements struck an optimal balance between accuracy and computational efficiency. This approach minimized accuracy loss while improving calculation efficiency by approximately 10%.

The loading point was achieved by coupling constraints between the set reference point and the loading surface of the midspan T-shaped plate. The support in the experiment was simply supported. The reference point was set at the model support. One end released constraints of the translation (U3) in the Z direction and the rotation (UR1) in the X direction to achieve sliding hinge bearing, and the other end released the rotation (UR1) constraint in the X direction to realize fixed hinge bearing. Finally, the displacement load was applied through the loading point RP-1 using automatic load steps.

### 4.2. Model Validation

**Figure 8:**
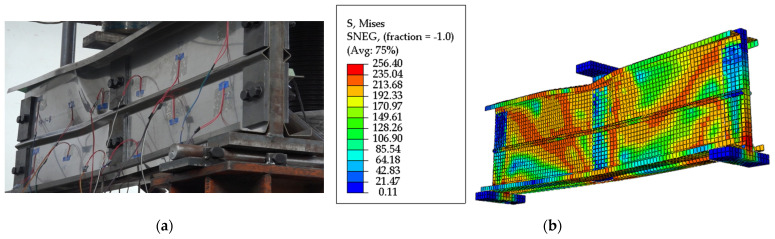
Verification of the failure characteristics of the model. (**a**) Experiment failure characteristics; (**b**) finite element failure characteristics.

The shear force–midspan vertical displacement curve and failure characteristics obtained from numerical simulation were compared with the experimental results. Figure 6 exhibits a comparison of shear force–vertical displacement curves between the experiment and finite element simulation. Due to the omission of the drift at bolted joints by the finite element model, the experimental results show a larger vertical displacement than the finite element results, with an error between the two of 10%, which indicates the reliability of the finite element analysis method. Figure 8 depicts the failure characteristics of the specimen. In Figure 8a, the load is controlled by displacement. At the beginning of loading, local buckling occurs at the flange, and the lateral displacement of the web expands. A tensile failure rising along a 45° diagonal line appears on the right web. As the load is added to around 100 KN, the diagonal tension failure extends to the upper web, the vertical deflection increases, and the failure continues to elongate, leading to distorted buckling of the beam flange. When the load reaches the ultimate value, the beam section is severely deformed. When the load drops to 80% of the ultimate bearing capacity, the loading is stopped, and the test ends. As shown in Figure 8b, the failure pattern of the finite element simulation is basically the same as that of the shear experimental specimen, both of which gradually undergo a 45° diagonal tensile failure of the web from the loading plate upwards, accompanied by the occurrence of flange distortion buckling.

Figure 6 implies that the shear force–vertical displacement curves of the experiment and finite element simulation are in good agreement. Figure 8 presents that the stress processes and failure characteristics of the two are consistent. It demonstrates that the established finite element model can effectively predict the shear capacity of the C-shaped beam with folded flanges.

This study only designed one experiment. Therefore, to further verify the correctness of the finite element method, the LCB beam proposed by Dissanayake et al. [45] was simulated using the same finite element method, and the simulation results were compared with corresponding experimental results, as shown in Table 2.

**Figure 9:**
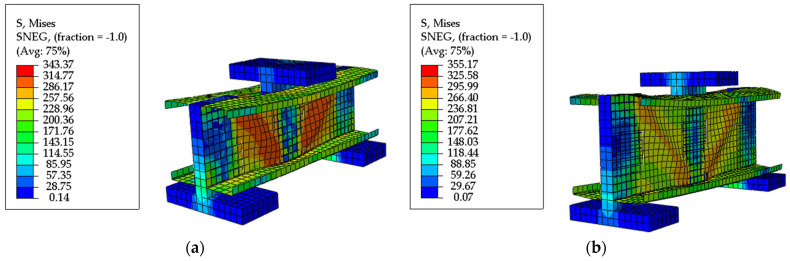
Finite element failure modes. (**a**) LCB 150 × 65 × 15 × 2.0; (**b**) LCB 200 × 75 × 15 × 1.2.

Table 2 shows that the average ratio of the numerical simulation results of the shear capacity of the specimen to the experimental observation results of Dissanayake et al. [45] is 0.988, with a variation coefficient of 0.071. Moreover, the failure mode obtained from the finite element simulation basically agrees with the experiment (as shown in Figure 5 in Reference [45]), as shown in Figure 9. Therefore, the finite element method in this study can be applied to a parametric analysis.

## 5. Influence Analysis of Finite Element Variable Parameters

In order to further understand the shear performance of C-shaped beams with folded flanges, this section conducts a parametric analysis after verifying the correctness of the finite element method in Section 4.2, which mainly includes the influences of the shear span ratio, complex edge stiffeners, web height–thickness ratio, and the size of the V-shaped stiffener size of the C-shaped beam with folded flanges.

### 5.1. Impacts of Shear Span Ratio and Complex Edge Stiffeners

The shear span ratio is a decisive factor in distinguishing the load-bearing characteristics of beams. When the shear span ratio is small, the beam is mainly controlled by the shear capacity. In order to investigate the influence of the shear span ratio on the shear capacity of the C-shaped beam with folded flanges, Table 3 lists three cross-sectional parameters under three shear span ratios for numerical simulation.

**Figure 10:**
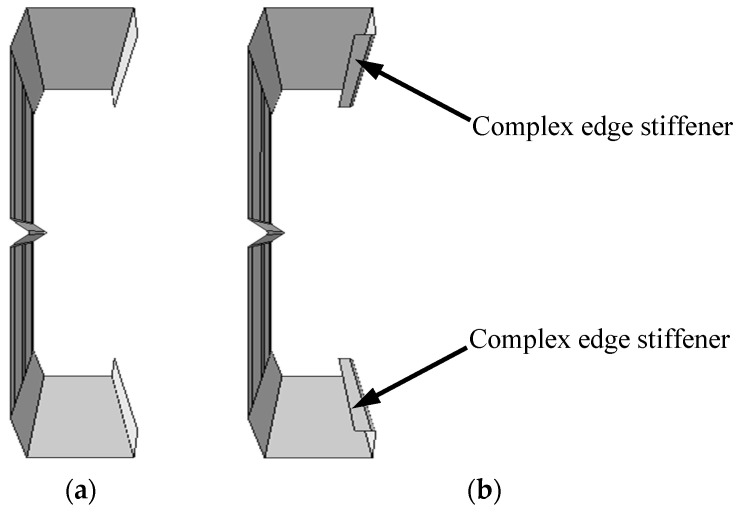
Schematic diagram of complex edge stiffeners. (**a**) Without complex edge stiffeners; (**b**) with complex edge stiffeners.

In addition, in practical engineering, the method of opening the web is mostly used to facilitate the installation of pipelines in steel beams. However, opening the web will inevitably affect its buckling performance. Therefore, steel beam sections with flanges and complex edge stiffeners have emerged, as shown in Figure 10. Generally, complex edge stiffeners can avoid the occurrence of flange distortion and buckling. However, global research mainly focuses on the bending capacity of complex edge stiffeners. This study explores the effect of complex edge stiffeners on the shear capacity of the C-shaped beam with folded flanges through finite element simulation. In the following components, K represents the shear span ratio A/H, and JB denotes the component with complex edge stiffeners.

Table 3 shows that when the shear span ratio is K = A/H = 1, the addition of complex edge stiffeners on the flange results in a significant upgradation (1.0%) in the shear capacity of the component with H = 220 mm; when the shear span ratio is K = A/H = 1.5, the addition of complex edge stiffeners on the flange leads to an obvious increase (0.5%) in the shear capacity of the C-shaped beam with H = 170 mm; and when the shear span ratio is K = A/H = 2, the addition of complex edge stiffeners on the flange causes a remarkable elevation (1.8%) in the shear capacity of the C-shaped beam with H = 170 mm.

**Figure 11:**
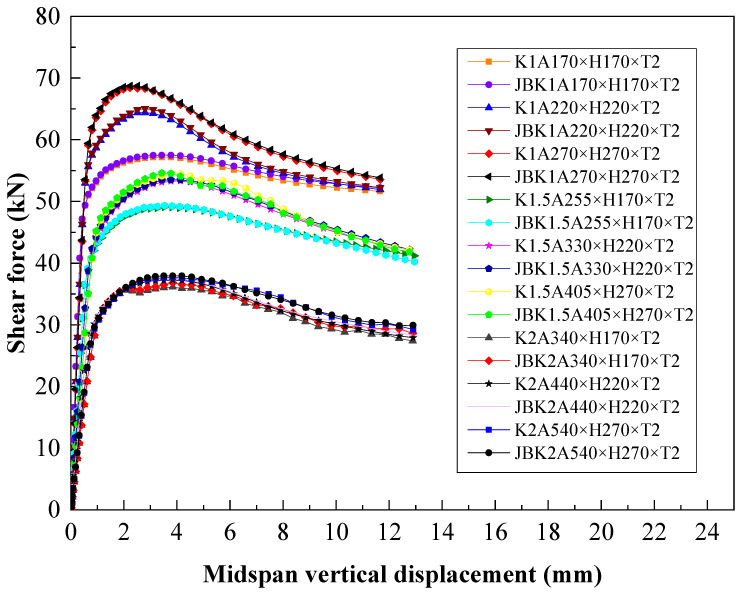
Effect of shear span ratio and complex edge stiffeners on bearing capacity and vertical displacement.

Figure 11 shows that for components with the same cross-section, as the shear span ratio increases, the midspan vertical displacement when reaching the ultimate load also grows, while the shear capacity and stiffness descend. However, when adding complex edge stiffeners to the component, there is no significant upward segment in the shear force–midspan vertical displacement curve, indicating that it has no profound improving effect on the shear capacity of the component.

### 5.2. Impact of the Web Height–Thickness Ratio

The web is the primary component that provides shear capacity for steel beams. To explore the effect of the height–thickness ratio of the web on the shear capacity of the C-shaped beam with folded flanges, a C-shaped beam with a shear span ratio of K = A/H = 1 was adopted for simulation to ensure that the shear force generated in the section is independent of the bending stress. Then, its shear performance was analyzed in depth.

**Figure 12:**
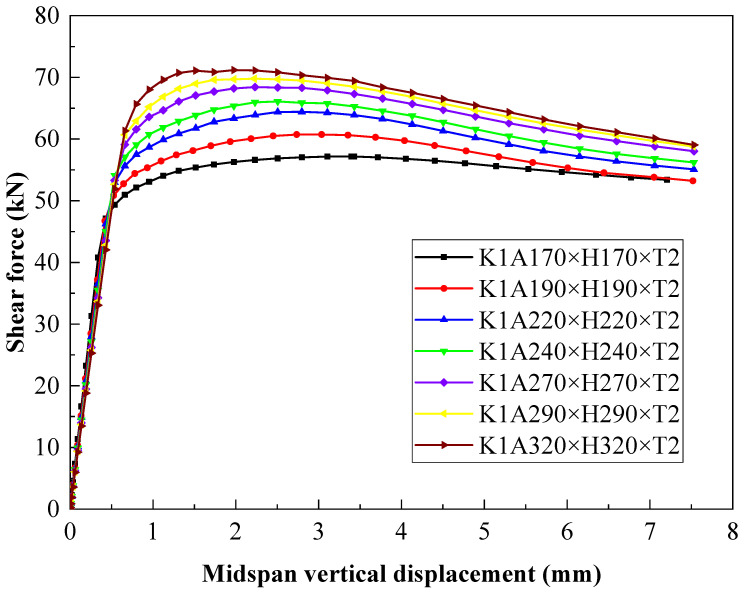
Effect of the web height–thickness ratio on bearing capacity and vertical displacement.

The seven specimens in Table 4 and Figure 12 have a shear span ratio of A/H = 1 and a thickness of T = 2. The web height–thickness ratios H/T are 85, 95, 110, 120, 135, 145, and 160, respectively. The finite element shear capacities are 57.18 kN, 60.73 kN, 64.40 kN, 66.09 kN, 68.44 kN, 69.78 kN, and 71.17 kN, and corresponding midspan vertical displacements are 3.43 mm, 3.04 mm, 2.80 mm, 2.51 mm, 2.23 mm, 2.22 mm, and 1.98 mm. These results indicate that when keeping the shear span ratio and thickness constant, as the height–thickness ratio increases, the shear capacity of the specimen is enhanced, and the midspan vertical displacement descends. The main reason is that the shear area of the web augments with the height–thickness ratio of the web, which promotes the shear capacity of the component. From the changing trend of the curve, the shear force–midspan vertical displacement curve of the C-shaped beam with a large web height–thickness ratio is located above, indicating that the stiffness of the component gradually rises, leading to a decline in the midspan vertical displacement.

The finite element ultimate load failure mode shows that when 135 ≤ H/T ≤ 160, the shear area of the web is relatively small, and the stress area mainly occurs at the loading point on the T-shaped plate. As the height–thickness ratio decreases, when 110 ≤ H/T ≤ 120, the stress begins to develop towards the middle of the web, the vertical displacement grows, and the flange begins to exhibit local buckling. When 85 ≤ H/T ≤ 95, the web plate undergoes diagonal buckling failure along the direction of 45°. Meanwhile, accompanied by distorted buckling of the flange, the midspan deflection further expands. The stress nephogram can intuitively interpret that the height–thickness ratio has a significant impact on the shear capacity of steel beams. Generally, the height–thickness ratio remarkably affects the shear capacity of steel beams and effectively elevates the overall stiffness within a specific range, abating the midspan deflection deformation of steel beams.

The study in Reference [32] proposed the direct strength method to calculate the shear capacity of LCB sections with web stiffeners. The formula (as shown in Equations (26) and (27) in Reference [32]) recommended in Reference [32] was used in this study to obtain the shear capacity of the C-shaped beam with folded flanges and intermediate stiffening webs. The calculation results were compared with the finite element simulation results. Table 4 shows that the average ratio of the numerical simulation results of the shear capacity of the specimen to the results of the direct strength method recommended in Reference [32] is 0.977, with a variation coefficient of 0.010. Therefore, this method can basically predict the shear capacity of the component in this study.

### 5.3. Effect of V-Shaped Stiffener Parameters

To obtain the effect of changing the parameters of the V-shaped stiffener on the shear capacity of the C-shaped beam with folded flanges, a C-shaped beam with a shear span ratio of K = A/H = 1 was employed for simulation. The simulation component was named K1A320 × H320 × T2–S*_b_* × S*_h_*–*θ*. The meanings of parameters are shown in Section 2.1.

**Figure 13:**
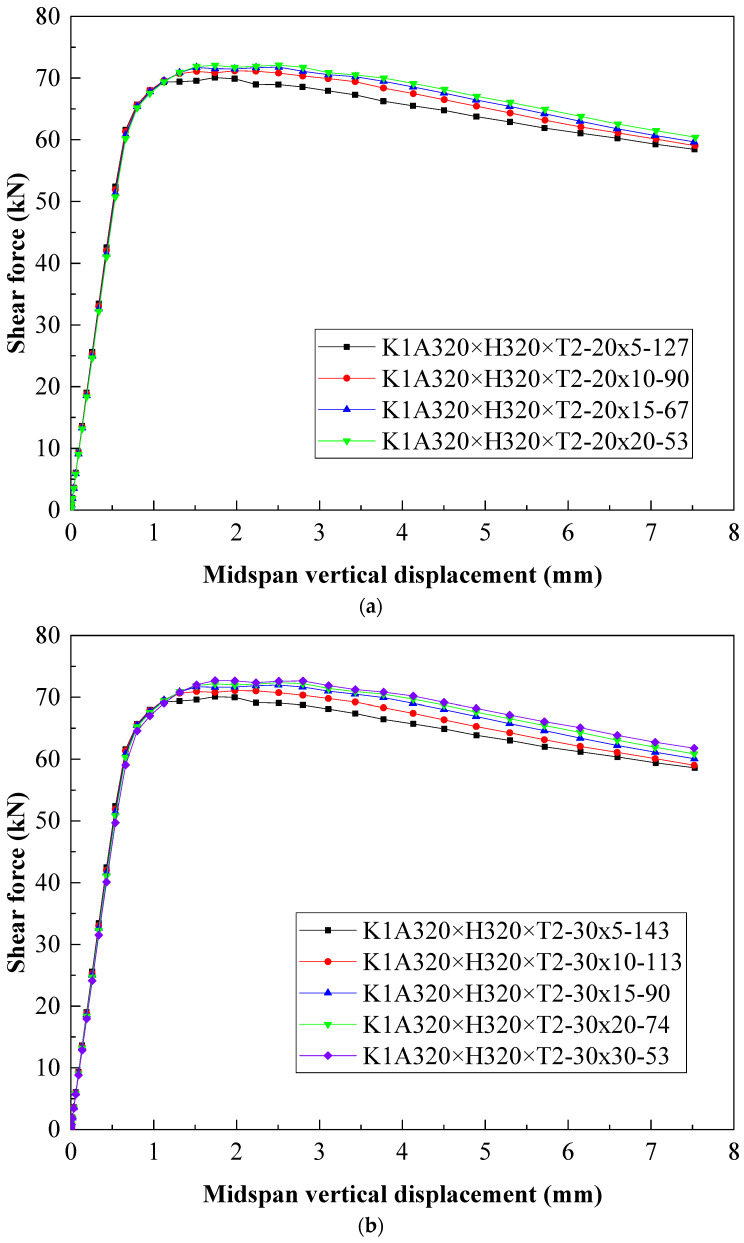

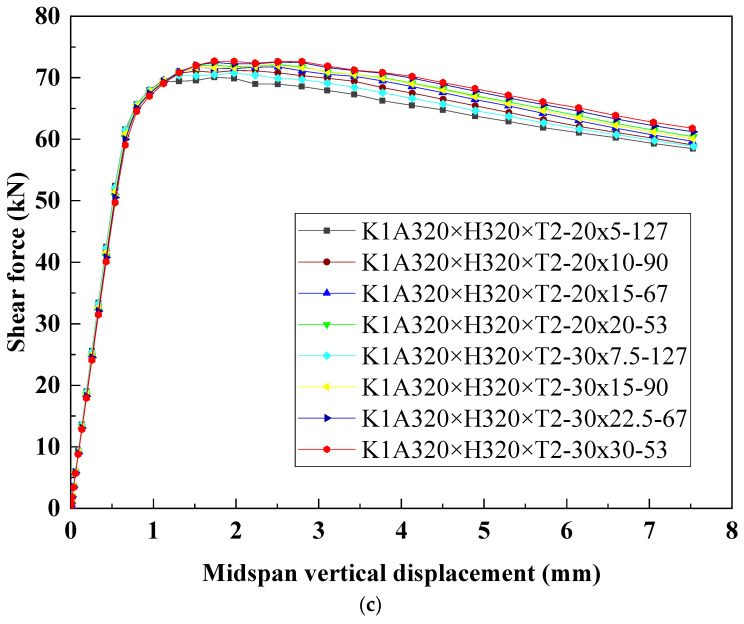
The influence of changing the parameters of the V-shaped stiffener on the bearing capacity and vertical displacement of the K1A320 × H320 × T2 beam. (**a**) S*_b_* = 20, (**b**) S*_b_* = 30, and (**c**) keeping θ constant, the effect of increasing S*_b_* and S*_h_*.

From Figure 13a,b, when the opening width S*_b_* of the V-shaped stiffener remains unchanged, with the increase in the height S*_h_* of the V-shaped stiffener, the shear capacity of the C-shaped beam slightly rises, the slope of the downward section of the shear force–midspan vertical displacement curve elevates, and the ductility of the component decreases. Figure 13c illustrates that when the angles between the two plates of the V-shaped stiffener are the same, as the width and height of the opening of the V-shaped stiffener increase, the ultimate bearing capacity of the C-shaped beam is mildly promoted, the slope of the descending section of the working curve rises, and the ductility of the component declines. The major reason is that a V-shaped stiffener is installed in the middle of the web of the C-shaped beam with folded flanges, which lowers the width–thickness ratio of the web and promotes the effective area of the C-shaped beam section under pressure, thereby advancing the bearing capacity of the C-shaped beam. As the angle between the two V-shaped stiffeners decreases, stress concentration becomes prominent, and ductility drops.

## 6. Conclusions

This study employs experiment and finite element simulation to investigate the shear performance of C-channel beams with folded flanges and mid-web stiffeners. The folded flanges and web stiffeners can both enhance the shear capacity of the member and mitigate the progression of local buckling. Consequently, this optimized cross-section can be widely applied to roof purlins and wall beams. The following conclusions are drawn:

(1) Through three-point bending tests on stainless C-channel beams with folded flanges and mid-web stiffeners, the impact of cross-sectional optimization on the ultimate bearing capacity and failure mode of the member was investigated. The folded flanges and web stiffeners help to retard the development of local buckling and improve the shear capacity of the member.

(2) The addition of complex edge stiffeners to the C-shaped beam with folded flanges cannot effectively advance the shear capacity of the component. The increase in the shear span ratio has a notable negative impact on the shear performance of the C-shaped beam. As the shear span ratio rises, the shear capacity of the components descends by 14–30%, the vertical displacement in the middle of the span increases, and the stiffness weakens.

(3) The height–thickness ratio of the web has a prominent impact on the shear performance of the C-shaped beam with folded flanges. The bearing capacity of the component is enhanced with the height–thickness ratio of the web, and the midspan deflection abates accordingly.

(4) When the width of the opening of the V-shaped stiffener and the angle between the two plates of the V-shaped stiffener remain unchanged, increasing the height of the V-shaped stiffener results in a small elevation in the shear capacity of the C-shaped beam and a decline in the ductility of the component. As thin-walled members are sensitive to initial imperfections, real-time measurement technology should be employed to monitor dimensional accuracy during the manufacturing of folded flange cross-sections. The tolerances for flange width, thickness, and folding angle should be controlled within ±1 mm, ±0.5 mm, and ±3°, respectively, while the length tolerance is generally maintained at around ±2 mm to ensure installation accuracy.

## Figures and Tables

**Table 1:** Measured geometric dimensions of specimens.

Specimen Number	*H*/mm	*F*/mm	*C*/mm	*D*/mm	*T*/mm	*S_b_*/mm	*S_h_*/mm	*θ*_1_/°	*θ*_2_/°
K2A640 × H320 × T2	322	28.85	48.45	19.25	2	20.05	9.55	106.75	96.50

**Table 2:** Comparison of the finite element shear capacity and experimental observations by Dissanayake et al. [45].

Specimen Number	Section/mm	*V*_Exp._/kN	*V*_FE_/kN	$\frac{V_{\mathrm{FE}}}{V_{\mathrm{Exp}.}}$
1	LCB 100 × 50 × 15 × 1.2	18.5	17.1	0.92
2	LCB 100 × 50 × 15 × 1.5	24.4	23.1	0.95
3	LCB 100 × 50 × 15 × 2.0	36.0	33.2	0.92
4	LCB 150 × 65 × 15 × 1.2	21.6	21.1	0.98
5	LCB 150 × 65 × 15 × 1.5	26.3	27.3	1.04
6	LCB 150 × 65 × 15 × 2.0	43.6	41.8	0.96
7	LCB 200 × 75 × 15 × 1.2	23.0	25.9	1.13
8	LCB 200 × 75 × 15 × 2.0	47.1	47.2	1.00
Mean				0.988
COV				0.071

**Table 3:** Finite element results of the shear span ratios and complex edge stiffeners.

Specimen Number	Section (mm)	Shear Span Ratio K = A/H	Complex Edge Stiffener Length (mm)	*V*_FE_/*V*_FE,JB_ (kN)	$\frac{V_{\mathrm{FE},\mathrm{JB}}}{V_{\mathrm{FE}}}$
1	K1A170 × H170 × T2	1	0	57.18	
2	JBK1A170 × H170 × T2	1	10	57.50	1.006
3	K1A220 × H220 × T2	1	0	64.40	
4	JBK1A220 × H220 × T2	1	10	65.04	1.010
5	K1A270 × H270 × T2	1	0	68.44	
6	JBK1A270 × H270 × T2	1	10	68.70	1.004
7	K1.5A255 × H170 × T2	1.5	0	49.08	
8	JBK1.5A255 × H170 × T2	1.5	10	49.32	1.005
9	K1.5A330 × H220 × T2	1.5	0	53.30	
10	JBK1.5A330 × H220 × T2	1.5	10	53.50	1.004
11	K1.5A405 × H270 × T2	1.5	0	54.54	
12	JBK1.5A405 × H270 × T2	1.5	10	54.58	1.001
13	K2A340 × H170 × T2	2	0	36.13	
14	JBK2A340 × H170 × T2	2	10	36.79	1.018
15	K2A440 × H220 × T2	2	0	37.24	
16	JBK2A440 × H220 × T2	2	10	37.49	1.007
17	K2A540 × H270 × T2	2	0	37.65	
18	JBK2A540 × H270 × T2	2	10	37.93	1.007

**Table 4:** Finite element results with different web height–thickness ratios.

Specimen Number	Section/mm	Shear Span Ratio K = A/H	Height–Thickness Ratio H/T	*V*_FE_/kN	Finite Element Ultimate Load Failure Mode	*V*_DSM_ [32]/kN	$\frac{V_{\mathrm{FE}}}{V_{\mathrm{DSM}}}$
1	K1A170 × H170 × T2	1	85	57.18	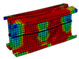	59.44	0.96
2	K1A190 × H190 × T2	1	95	60.73	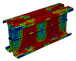	61.97	0.98
3	K1A220 × H220 × T2	1	110	64.40	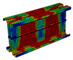	65.24	0.99
4	K1A240 × H240 × T2	1	120	66.09	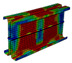	67.16	0.98
5	K1A270 × H270 × T2	1	135	68.44	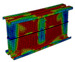	69.75	0.98
6	K1A290 × H290 × T2	1	145	69.78	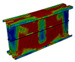	71.32	0.98
7	K1A320 × H320 × T2	1	160	71.17	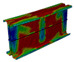	73.47	0.97
Mean							0.977
COV							0.010

## Data Availability

The original contributions presented in this study are included in the article. Further inquiries can be directed to the corresponding authors.
